# Networks through the lens of high-frequency oscillations

**DOI:** 10.3389/fnetp.2024.1462672

**Published:** 2024-11-28

**Authors:** Aline Herlopian

**Affiliations:** Yale Comprehensive Epilepsy Center, Department of Neurology, Yale School of Medicine, New Haven, CT, United States

**Keywords:** high-frequency oscillations, ripples, fast ripples, network, propagation, connectivity, biomarker, cluster

## Abstract

To date, there is no neurophysiologic or neuroimaging biomarker that can accurately delineate the epileptogenic network. High-frequency oscillations (HFO) have been proposed as biomarkers for epileptogenesis and the epileptogenic network. The pathological HFO have been associated with areas of seizure onset and epileptogenic tissue. Several studies have demonstrated that the resection of areas with high rates of pathological HFO is associated with favorable postoperative outcomes. Recent studies have demonstrated the spatiotemporal organization of HFO into networks and their potential role in defining epileptogenic networks. Our review will present the existing literature on HFO-associated networks, specifically focusing on their role in defining epileptogenic networks and their potential significance in surgical planning.

## 1 Introduction

The evolution in the conceptualization of epilepsy as a network pathology propelled the endeavor to identify neurophysiological and neuroimaging biomarkers to delineate and map epileptogenic networks. High-frequency oscillations (HFO) are considered biomarkers of epilepsy (Jacobs et al., 2008; Frauscher et al., 2017; Jacobs et al., 2012; Sharifshazileh et al., 2021; Burelo et al., 2021). They are associated with epileptogenesis and ictogenesis (Zijlmans et al., 2012; Roehri and Bartolomei, 2019; Roehri et al., 2018; Li X. et al., 2021; Bragin et al., 2004; Bragin et al., 2007; Santana-Gomez et al., 2019; Jiruska et al., 2010; Xu et al., 2016; Ferrari-Marinho et al., 2016). High-frequency oscillations are present at high rates in ictal onset areas and “epileptogenic zones,” and their resection is associated with favorable postoperative outcomes (Höller et al., 2015; Burnos et al., 2016; Amiri et al., 2016; Zijlmans et al., 2011; Worrell et al., 2004; Chen et al., 2021; Cho et al., 2014; Jacobs et al., 2010; Akiyama et al., 2011; van't Klooster et al., 2015; van Klink et al., 2014; Fujiwara et al., 2012; Wu et al., 2010; Jiruska et al., 2017).

Epileptogenic networks are conceptualized to have a hierarchical organization, ranging from microscale circuits to macroscale networks (Zaveri et al., 2020; Barot, 2020). To study epileptogenic networks on a smaller scale or at the level of local neural circuitry, it is essential to investigate HFO (Barot, 2020). Local microscale networks, comprising pyramidal cells and interneurons, generate HFO (Shamas et al., 2018; Jefferys et al., 2012; Helling et al., 2015; Righes Marafiga et al., 2021; Shiri et al., 2016). Physiological and pathological ripples (R, 100–250 Hz) and fast ripples (FR, 250–500 Hz) are generated by a variety of neural microcircuits through mechanisms that can be distinct, overlapping, or, in certain cases, similar (Bragin et al., 2007; Ylinen et al., 1995; Bragin et al., 2011; Lai et al., 2023; Liu et al., 2022; Buzsáki, 2015). Therefore, it is reasonable to conceptualize HFO as small-scale networks that evolve into larger-scale networks.

Initially, HFO were considered spatially restricted without the ability to “propagate” or “organize within networks or highly connected clusters” (Cuello-Oderiz et al., 2017; van Diessen et al., 2013a). Micro- and macro-electrode recordings, non-invasive studies, and connectivity analyses support the presence of “HFO hubs,” “HFO networks,” and “HFO propagation” (van't Klooster et al., 2015; van Klink et al., 2014; van Diessen et al., 2013a; González Otárula et al., 2019; Xu N. et al., 2021; Ortiz et al., 2018; Korzeniewska et al., 2014; Zweiphenning et al., 2016; Weiss et al., 2022; Weiss et al., 2019). These findings raise the question of whether surgical failure risk can be mitigated by adequate disconnection of “critical” HFO-generating networks or “HFO hubs.”

Isolated HFO, “HFO hubs,” and “HFO networks” exhibit dynamic changes comparable to those observed in epileptogenic networks, including changes associated with arousal states and pre-ictal and post-ictal phases (Worrell et al., 2004; Halász et al., 2019; Staba et al., 2004; von Ellenrieder et al., 2017; Bagshaw et al., 2009; Halász and Szűcs, 2020; Song et al., 2016; Epstein et al., 2014; Inoue et al., 2020; Fisher et al., 1992; Zweiphenning et al., 2019; Birk et al., 2021). Despite this variability and dynamic nature, the localization and morphology of the “HFO networks” remain constant and centered around the foci that initiate seizures (Fisher et al., 1992).

This review addresses our understanding and knowledge gaps regarding “HFO networks” and “HFO hubs,” their spatiotemporal organization, correlation with foci initiating seizures, and association with surgical outcomes. The presented evidence substantiates the importance of multimodal investigation, integrating neuroimaging data, electrophysiological recording, and connectivity measures in studying “HFO networks,” “HFO hubs,” and “HFO clusters.”

## 2 Mechanisms of HFO generation from cellular to network levels

The generation of HFO involves myriad mechanisms (Jiruska et al., 2017; Jefferys et al., 2012). A single neuron cannot solely and sufficiently generate HFO over a broad spatial range, seen as a prominent spectral power peak corresponding to a focal EEG event (Jefferys et al., 2012). Intrinsic cellular and synaptic characteristics are essential to generate and sustain oscillations in the HFO range, particularly FR, at a neural network level. One proposed mechanism is principal cell action potentials or synchronized inhibitory postsynaptic potentials with sparse pyr with sparse pyramidal cell firing (Jiruska et al., 2017; Jefferys et al., 2012). The synchronized fast firing of these interconnected neurons leads to the formation of high-frequency spikes, recorded as HFO.

An alternative hypothesis is the “out of phase” theory for pathological HFO generation, particularly FR. Multiple subpopulations of synchronized cluster neurons fire with phase delays or lag. This results in a net FR frequency band for these subpopulations that fire as individual cluster units at lower frequencies with phase lag (Jiruska et al., 2017; Jefferys et al., 2012). The complex network connectivity patterns of the “hub neurons” and the distinct populations of cell clusters, primarily the principal cells and the interconnected neural populations, influence the functionally “out of phase” firing and, ultimately, the generation of FR (Jiruska et al., 2017; Santana-Gomez et al., 2022). The phase difference may be secondary to the complex spatiotemporal interaction of various current dipoles in the extracellular field, leading to the formation of population HFO spikes (Jefferys et al., 2012). Firing of these neurons at a specific latency can contribute to the same spike event (HFO) population; otherwise, an independent HFO (particularly FR) cycle may arise if the firing occurs at a later point in time. This phenomenon elucidates the non-synaptic mechanism by which functionally similar neuronal clusters generate HFO, primarily FR. Other potential mechanisms for generating FR include the asynchronous firing of diverse networks of neurons consisting of highly active interneurons and pyramidal cells (Jiruska et al., 2017).

Furthermore, the morphological alteration of neurons due to increased extracellular glutamate levels, high intracellular calcium levels, deregulation of the signaling pathway, and organelle dysfunction can lead to cell loss, subsequent axonal sprouting, and activity-dependent synaptic reorganization (Santana-Gomez et al., 2022). These morphological neuronal changes alter the connectivity pattern, reinforce excitatory connections between neurons, and form pathologically interconnected neurons (PIN). These PIN are hypothesized to generate hypersynchronous cyclic bursts of population spikes that are pathologically robust, as evidenced by HFO recordings in the 200–300 Hz range. These PIN indicate neuronal disturbances, and their development is linked to epileptogenesis and epilepsy (Santana-Gomez et al., 2022; Bragin et al., 2000; Li L. et al., 2019). These topologically different clusters of neurons can be recruited “out of phase” with varying lags, leading to the generation of HFO (Jefferys et al., 2012). In animal model studies with an episode of status epilepticus caused by an intra-hippocampal kainic acid-induced lesion and traumatic brain injury, PIN clusters are formed at local sites and at locations that are distant from the induced-lesioned areas (Li L. et al., 2019; Li L. et al., 2021). Consequently, these focal PIN clusters create widespread PIN and increase in the HFO rates. Similarly, pathological HFO can induce the emergence of remote HFO, and along with cross-frequency coupling, they can contribute to epileptogenesis (Gelinas et al., 2016).

## 3 Recording HFO at the cellular and network levels

Various types of intracranial contacts are used to record HFO (Wang et al., 2020; Bragin et al., 1999). Hybrid micro-macroelectrode contacts, consisting of conventional clinical macroelectrodes typically used for intracranial investigation as part of surgical workup in conjunction with microwires at the tip of each depth electrode for research purposes, are commonly used to record HFO (Worrell et al., 2008).

In contrast to macroelectrodes, which provide more comprehensive anatomical coverage, microelectrodes have more restricted spatial coverage for recording single-unit activity (Wang et al., 2020). Microelectrodes capture a broader spectrum of frequency ranges (R, FR, and ultrafast ripples) than macroelectrodes, leading to greater number of HFO events (Wang et al., 2020; Bragin et al., 1999; Worrell et al., 2008). This is primarily due to the focal sampling advantage of microelectrodes, making them particularly advantageous for capturing HFO, particularly FR, which are typically microscale events with a very local propagation or spatial distribution. Hence, microelectrodes are more suitable than macroelectrodes for capturing HFO, particularly FR (Worrell et al., 2008; Barth et al., 2023; Curot et al., 2023; Despouy et al., 2019; Despouy et al., 2020; Chari et al., 2020). The spatiotemporal distribution of FR is quantified through hybrid recording, and its spatial scales are quantitatively distinct from R (Worrell et al., 2008). This observation elucidates why FR are present on a single macroelectrode contact but absent on the immediate neighboring contacts, raising questions about the clinical implications of this phenomenon on surgical planning (i.e., undersampling of the macroelectrodes in contrast to microelectrodes).

The design of microelectrodes has evolved from 1 mm to 0.05 mm spacing to enhance the recording of the very focal HFO events (Worrell et al., 2008; Chari et al., 2020; Blanco et al., 2011; Yang et al., 2021). These microelectrodes record multiscale single-unit or multiunit neuronal activity with improved spatial resolution and signal visualization (Worrell et al., 2008; Despouy et al., 2019; Despouy et al., 2020; Chari et al., 2020; Blanco et al., 2011; Yang et al., 2021; Kondylis et al., 2014). To improve the recording of HFO events that are not present in conventional hybrid recordings, current hybrid micro-macroelectrodes utilize tetrodes, which are bundles of microwires, rather than a single microwire neural electrode (Curot et al., 2023; Despouy et al., 2019; Despouy et al., 2020). Recordings from tetrodes confirms the exceedingly confined nature of HFO, especially FR. Novel microelectrocorticographic arrays are employed for intraoperative recording, confirming that macroelectrodes capture only about 44% of the HFO events recorded when using the novel arrays (Barth et al., 2023). These innovative arrays can capture HFO on single, multiple, contiguous, and non-contiguous microcontacts.

## 4 Analysis of HFO

The computational analysis of HFO entails several steps. Initially, data acquisition is conducted. This is followed by data pre-processing (such as EEG data extraction, frequency filtering, signal thresholding, wavelet transformation, and template matching). Next, post-processing data analysis is conducted, encompassing spatial mapping and clustering of HFO events, spectral analysis of HFO features, and visualization of the topographic distribution of HFO to discern physiological and pathological features. Ultimately, data are interpreted to contextualize the findings within a clinical framework.

A critical step in the pre-processing phase is data extraction. Initially, select the EEG epoch using the institutional native EEG reader software. Typically, multiple epochs of 5–10 min or a single epoch of 15–30 min are selected, ensuring they are at least 1 hour (typically 4–6 h) away from seizures, as the rates and characteristic features of HFO vary according to their proximity to the ictal period (Malinowska et al., 2015; Pearce et al., 2013). The chosen epochs should include periods with minimal artifacts and exhibit the highest prevalence of HFO, namely, during slow-wave sleep or non-rapid eye movement (NREM) rather than during wakefulness or REM states (Frauscher et al., 2017; von Ellenrieder et al., 2017; Bagshaw et al., 2009; Gliske et al., 2018; Dümpelmann et al., 2015). After clipping the EEG epoch (video may be removed at this point or during export), it is exported and converted to. edf format using either native reader conversion software, other EDF conversion tools, or Python/MATLAB scripts, ensuring a de-identified format for data pre-processing.

Following data extraction, HFO detection is accomplished through visual annotation, automated detection, or a combination of automated detection followed by visual validation. Although visual annotation reduces false positive detections (e.g., artifacts), it is highly time-consuming and lacks reproducibility due to poor inter-rater agreement; in contrast, automated detectors exhibit more detections with higher false positive rates but are more reproducible and efficient (Roehri and Bartolomei, 2019; Chen et al., 2021; Gliske et al., 2018; Burnos et al., 2014; Ren et al., 2019; Charupanit et al., 2018; Jacobs et al., 2009; Spring et al., 2017; Nariai et al., 2018; von Ellenrieder et al., 2012; Staba et al., 2002; Blanco et al., 2010; Liu et al., 2018; Thomas et al., 2023). That said, the predominant methodologies for visual detection encompass the following steps (Burnos et al., 2016; Li et al., 2022; Wang et al., 2022; Zelmann et al., 2009; Worrell et al., 2012; Zelmann et al., 2012; Ye et al., 2024; Remakanthakurup Sindhu et al., 2020; Sindhu, 2023).

1. Implement the desired bandpass filter parameters (e.g., LFF = 250 Hz and HFF = 500 Hz for FR) and expand the window to 0.5 and 0.8 s.
2. Identify a singular HFO event by discerning distinct oscillations from the interictal baseline background activity. Note that there is no definitive threshold for percent change in amplitude or amplitude difference between baseline interictal background activity and HFO event amplitude. Also, there is no consensus regarding HFO signal onset and offset. Nevertheless, an amplitude range of 10–1,000 µV is used to identify an HFO event.
3. Select a single HFO event with at least four oscillations (some centers utilize three oscillations), distinguishing it from background activity. The duration of HFO events varies, with R ranging from 100 to 1,000 milliseconds and FR from 30 to 50 milliseconds, as do their frequency band ranges.
4. Measure the interevent duration, which must be a minimum of 25 milliseconds; two HFO events are considered independent if the interval difference is at least 25 milliseconds.
5. Recognize the presence of various HFO morphologies, including regular (consistent frequency band range and static amplitude) and irregular (a combination of R and FR frequencies with variable amplitude peaks).

Many automated detectors use MATLAB (RIPPLELAB, HFOApp, EPINETLAB), Python (MNE-HFO), and EEGLAB algorithms (MATLAB-based) (Zhou et al., 2022; Navarrete et al., 2016; Quitadamo et al., 2018; Zhang et al., 2024a). Many academic and research-based institutions use their own automated detectors, which are coded with parameters that they deem critical (von Ellenrieder et al., 2017; von Ellenrieder et al., 2012). Automated detector development necessitates advanced programming skills and the input of clinical epileptologists to ensure that the detection parameters generate clinically relevant data rather than just statistically, mathematically, or algorithmically significant data. Automated detector algorithms should include parameters such as threshold (establishing cut-off values to accept a specific oscillation burst as a detection), minimum duration of event, minimum time between events, window size for the root mean square (RMS) amplitude, minimum number of peaks or oscillations, and other findings that may be critical to minimizing false positives (Mendoza et al., 2023). Please be advised that certain other parameters (such as polarity, slope of oscillations, and latency in the propagation of HFO) are critical; however, they may not be included in these automated detectors.

One of the most important questions is how to choose the most suitable automated detection algorithm (Charupanit et al., 2020). Currently, no single automated detection tool is the most reliable. Maltseva et al. conducted a comparison of 11 automated detectors by analyzing 30-min epochs from the N2 sleep state of 15 patients who were undergoing intracranial and scalp EEG recordings (Maltseva et al., 2023). The detectors did not demonstrate superior performance in their original recording methods (e.g., developed for scalp use and later employed for intracranial detection). In general, the detections from scalp and intracranial EEG studies were comparable, with no detector exhibiting superiority over the others. The agreement was substantial when identifying areas with higher HFO rates, despite the low inter-rater agreement when identifying individual HFO events.

As an alternative to the previously stated points, some centers advocate for the simultaneous use of multiple automated detectors given their comparable performance to increase the likelihood of accuracy and reduce the number of false positives (Zelmann et al., 2012; Maltseva et al., 2023). Meanwhile, other centers perform visual validation after automated detection to ensure high specificity and eliminate false positives.

Deep machine learning algorithms and models serve as an alternative for detecting HFO and analyzing their characteristics, dynamic patterns, and spatiotemporal distribution (Zhou et al., 2022; Navas-Olive et al., 2022; Navas-Olive et al., 2024; Shoeibi et al., 2021; Nadalin et al., 2021; Zhao et al., 2020; Zuo et al., 2019; Xu Z. et al., 2021; Hagen et al., 2021; Zeimarani et al., 2020; Amiri et al., 2016). These algorithms and models offer enhanced accuracy in characterizing HFO and demonstrate reliability in artifact rejection. They can be trained to replicate expert labeling and differentiate between various HFO based on specific features, thereby enhancing understanding of the distinctions between physiological and pathological states and their associations with cognitive tasks, epileptic networks, and surgical outcomes. Machine learning encompasses various algorithms and models, including convolutional neural networks (CNNs), recurrent neural networks (RNNs), and long short-term memory (LSTM) networks.

Deep machine learning algorithms enhance HFO detection via two methodologies: supervised and unsupervised training. These models necessitate less rigorous manual engineering features compared to automated detector development. They enhance the efficiency of analyzing complex and large datasets. In supervised learning models, including neural networks, linear regression, decision trees, and support vector machines, annotated or labeled datasets, referred to as “input data” (e.g., tracings marked with HFO and non-HFO events), are provided to the algorithm to train the model based on the identified/labelled features of the events (output data). Upon learning the relationship between the input and output data, the model extracts and utilizes these identified features to generate predictions for unknown datasets. Subsequently, the model is subjected to validation and testing with a new set of HFO data. Ma et al. employed a supervised CNN algorithm to train the model to identify HFO. The model demonstrated its ability to effectively identify HFO, surpassing traditional detection methods in terms of detection accuracy and minimizing false positives (Ma et al., 2019).

Meanwhile, unsupervised learning (including autoencoders and clustering algorithms) involves training a model on unlabeled data. This approach enables the model to discern intrinsic or hidden features and patterns without explicit feedback, thereby learning from the fundamental patterns present in the data.

Zhang et al. utilized a reverse engineering approach to analyze labeled HFO events on intracranial EEG (Zhang et al., 2022a). Their model demonstrated ability to differentiate HFO features associated with epileptogenic and non-epileptogenic regions. In another study, Zhang et al. employed a weak supervision approach to characterize the morphological features of physiological HFO (Zhang et al., 2022b). Their model demonstrated high accuracy and efficacy in distinguishing between various types of physiological HFO compared to conventional methods. The model learned the pertinent features of the raw data, eliminating the need for manual feature extraction. Validation was performed on an independent dataset to confirm the model’s capacity to generalize to new and unlabeled data. The approach markedly decreased false positives relative to traditional methods.

Variational autoencoders represent another example of a learning model (Higgins et al., 2021). Zhang et al. conducted a multi-center study involving 185 patients with epilepsy who underwent intracranial investigation (Zhang et al., 2024b). A total of 686,410 HFO were analyzed and classified into three categories: a) morphologically defined putative pathological HFO (mpHFO), b) morphologically defined putative non-pathological HFO, and c) morphologically defined artifacts of extracerebral origin. The study indicated that mpHFO were associated with spike and originated from seizure onset zones. The removal of mpHFO demonstrated a greater predictive value for postoperative seizure freedom compared to the resection of the seizure onset zone alone. The HFO originating from the seizure onset zone exhibited similar characteristics across different anatomical regions, in contrast to the physiologic HFO, which displayed distinct features compared to HFO from seizure onset zones.

Machine learning models have similarly advanced the understanding of hippocampal sharp-wave ripple (SWR) events (Navas-Olive et al., 2022; Navas-Olive et al., 2024; Nadalin et al., 2021; Kulkarni et al., 2019; Aizpurua et al., 2016). These models enable the differentiation of physiological SWR during cognitive tasks from pathological events by facilitating the visualization and clustering of input data. This approach aids in examining the interactions among various types of R that emerge during memory processing. Large datasets were analyzed with clustering algorithms to categorize hippocampal HFO according to various neural states, such as sleep-related SWR *versus* hippocampal FR (Sebastian et al., 2023). Therefore, the topological analysis of these SWR will contribute to understanding their function in memory processing and how pathological FR in the hippocampi can disrupt physiological neural activity.

It is still uncertain which machine learning algorithm and model to use in the analysis of HFO, as well as the appropriate format for the “input data,” similar to automated detectors. Although visual annotation or visual validation of automated detections is considered the current gold standard, data augmentation is a novel approach proposed to address the challenges encountered when using machine learning algorithms and models due to the heterogenous nature of the limited number of datasets. Data augmentation involves artificially enhancing and expanding the original dataset, thereby increasing the “synthetic” number of HFO events available for additional neural network training (Schlafly et al., 2024). This technique can assist in training deep machine learning algorithms to detect HFO with high precision and reliability, while enhancing the reproducibility of analyses (Nariai et al., 2018; Hagen et al., 2021; Schlafly et al., 2024; Boran et al., 2019; Li XHY. et al., 2019; Korshunova et al., 2017; Dubey et al., 2022; Rasheed et al., 2021; Wei et al., 2019; Medvedev et al., 2019).

## 5 Invasive recording of HFO

### 5.1 Intracranial monitoring (subdural contacts and depth electrodes)

Most of the data regarding HFO are derived from intracranial recordings. However, over the years, intracranial EEG (iEEG) studies have been paired with non-invasive (e.g., magnetoencephalography, MEG) and invasive (e.g., single pulse electric stimulations, SPES) modalities to overcome the sampling limitations of iEEG and to provide a more spatial context for HFO analysis. The data from iEEG indicates that FR are localized events closely associated with epileptogenic zones and seizure onset areas. Unlike non-invasive modalities, FR are readily captured through intracranial recording (Jacobs et al., 2010; Akiyama et al., 2011; Schevon et al., 2009). In contrast, R are more widespread events that can be easily recorded using invasive and non-invasive methods (von Ellenrieder et al., 2016a; Tamilia et al., 2020). Recently, HFO greater than 500 Hz have been recorded; they are spatially very focal pathological phenomena, significantly more restricted than FR and R (Hao et al., 2021; Brázdil et al., 2023). They are hypothesized to be better biomarkers for epileptogenic zones. Reduced or weakly coupled networks in the epileptogenic tissue can generate very high-frequency (500–1000 Hz) or ultra high-frequency (>1000 Hz) HFO (Přibylová et al., 2024).

Initial data from iEEG explored HFO as solitary events and revealed an association between interictal HFO rates and seizure onset zones (Jacobs et al., 2008; Zijlmans et al., 2012; Ferrari-Marinho et al., 2016; Wu et al., 2010). Nevertheless, HFO are not static phenomena at a given focus; instead, they propagate or exhibit organization, distribution, or clustering throughout the brain (González Otárula et al., 2019; Korzeniewska et al., 2014; Jahromi et al., 2021; Tamilia et al., 2018; Song et al., 2024). High-frequency oscillations form a sequential pathway on iEEG recordings by traversing various cortical regions (distribution contingent upon frequency band) within a very brief timeframe (González Otárula et al., 2019; Tamilia et al., 2018; Cai et al., 2021).

The term “HFO propagation” has been extensively used in the literature to denote the sequential occurrence of HFO at any frequency range across various contiguous and non-contiguous contacts within a specified timeframe. Jahromi et al. described HFO as having onset contacts (displaying the first HFO event) or spread contacts (displaying HFO events lagging the onset contact) based on their temporal involvement in the propagating sequence (Jahromi et al., 2021). They demonstrated that propagating HFO patterns exhibited a common HFO onset region, determined to be a predictor of seizure onset and resection zones. The entire HFO propagation zone had higher sensitivity in predicting the resection zone than individual HFO events.

Song et al. observed that interictal HFO fired at significantly higher rates in the seizure onset zone than in the non-seizure onset areas in patients with refractory mesial temporal lobe epilepsy (Song et al., 2024). In their cohort, seizures arose from the amygdala and propagated to the hippocampus. The amygdala and hippocampus had higher HFO rates interictally than the lateral temporal neocortex. The HFO were coupled with theta oscillations, significantly more in the amygdala, hippocampus, and, to a lesser extent, the lateral temporal neocortex. During the immediate pre-ictal state, HFO rates increased in the amygdala, with a significantly high coupling rate between HFO and theta oscillations. During the ictal phase, the HFO firing rate increased dramatically in the hippocampus compared to the amygdala and the lateral temporal neocortex. Their findings revealed the dynamic nature of interictal-ictal HFO, mirroring the ictal onset and early propagation pathways. Furthermore, they proposed the coupling of interictal HFO with theta oscillations as a potential biomarker for the seizure onset zone.

Otarula-Gonzalez et al. described HFO network characteristics (González Otárula et al., 2019). These networks were defined by the temporal sequence of HFO events, displaying variable latencies between contact pairs. Each patient had more than one independent R-network but only one FR-network. The R-networks were longer, while the FR-networks were shorter. The source contacts in the R-network were variable and had more contacts than the FR-networks, which had limited source contacts. The earliest contact in the network had higher R and FR rates than the farthest, which had the lowest rates. The initial source contact was resected more frequently than the remaining source and non-source contacts of the R- and FR-networks. Nonetheless, all source and non-source contacts of the FR-networks were restricted to the seizure onset zone, unlike the R-networks. There was no difference in the proportion of the resected source channels and the non-source channels for both R and FR. However, the first source channel was resected in a similar ratio to the channel with the highest HFO rate because the first source channel often corresponded to the channel with the highest HFO rate. The investigators concluded that the resection of the first source channel was not superior to the resection of channels with the highest HFO ratio.

Cai et al. demonstrated that HFO sequences exhibited a highly consistent and repeatable propagation pattern (repetitive instances of similar electrode contacts within the same patient) across various organizational HFO clusters (Cai et al., 2024). These HFO clusters had a variety of spatiotemporal pathways across patients that engaged various cortical regions over extended periods. Some channels exhibited a bimodal pattern of sequential order within a given HFO network/sequence, with instances of either leading or lagging, suggesting variability in the recruitment order within a given HFO sequence. The HFO recruited core, defined as contacts of propagation, had a significant overlap with the seizure onset zone.

Furthermore, higher HFO rates and more prevalent HFO sequences were noted in seizure onset zones than in non-seizure onset areas. However, the entire HFO sequence rather than HFO rate had predictive power in identifying the seizure onset area and epileptogenic zone and predicting surgical outcomes (Cai et al., 2024). Additionally, the epileptogenic zone was more precisely defined by the entire HFO sequence, including the propagation pathway, as opposed to the HFO onset contacts alone within a given sequence. This observation was contrary to the finding in Tamilia et al.‘s study where the R onset zone was critical for alignment with the seizure onset zone as opposed to the entirety of the R zone (Ren et al., 2019). While 40% of the HFO sequences originated outside or near the seizure onset zone, even in seizure-free patients following resection, unfavorable outcomes were noted when the clinically defined seizure onset zone corresponded to the channels that were recruited later in the HFO sequence, underscoring the importance of the entirety of the HFO sequence in delineating the area initiating seizures (Cai et al., 2024). This implied that the ictogenic focus extended beyond the traditionally defined seizure onset zone in intracranial studies. The findings in Cai et al.’s study emphasized that the closer the whole HFO sequence aligned spatially with the clinically presumed epileptogenic zone, the higher the likelihood of attaining seizure freedom.

Moreover, connectivity analyses confirmed the presence of initiation and suppression mechanisms for HFO propagation (Cai et al., 2024). The connectivity measures differed in regions involved in HFO sequences with overlapping seizure onset zones compared to the peripheral non-seizure onset areas. There was a high synchrony of oscillations in the regions initiating HFO sequences, coupled with a prominent inward information flow from the periphery to the HFO core zones to halt and suppress HFO propagation. There was a noticeable increase in coherence within the seizure onset zone and the HFO zone compared to the periphery (non-seizure onset foci), implying elevated levels of synchronization between the HFO zone and the seizure onset zone during propagation.

The findings above underscore the significance of pivoting from analyzing HFO as isolated events to probing their dynamic spatiotemporal organization in relation to epileptogenic networks and lesions. The ultimate goal is to integrate these findings into the surgical decision-making process.

### 5.2 Intraoperative electrocorticography

The predictive value of HFO in determining postoperative outcomes has been explored in numerous studies using intraoperative electrocorticography (iEcog) (van 't Klooster et al., 2017a; Hussain et al., 2017; Hussain et al., 2016; van Klink et al., 2021; Peng et al., 2021). Resection of FR was highly predictive of postoperative seizure outcome in a pediatric cohort with lesional epilepsy followed for a median of 4 years (Hussain et al., 2017; Hussain et al., 2016). Incomplete resection of the FR was associated with seizure recurrence within 6 months. The superiority of HFO over epileptiform discharges in guiding resection and predicting surgical outcomes was demonstrated by van 't Klooster et al. (2015). Furthermore, removing the distant pre-resection HFO was not considered necessary for improving postoperative outcomes. The investigators believed that distant HFO were secondary to propagation from HFO foci that were present pre-resection and abolished with disconnection of the epileptic network.

van Klink et al. (2014) displayed that the resection of R, R on spikes, and spikes did not impact the outcome. However, only pre-resection FR, with twice as many FR in the resected area compared to the non-resected areas, was associated with a favorable outcome. The study’s intriguing finding was the *de novo* emergence of R in the sensorimotor cortex in patients with good outcomes, which was likely attributed to the disconnection from the epileptogenic zone through resection, leading to the functional release of the dependent sensorimotor area and regaining functionality, with the *de novo* emerging R possibly serving as a neurophysiological biomarker. van’t Klooster et al. determined that the presence or absence of post-resection FR, rather than the percentage of resection of pre-resection FR and R, was a predictor of postoperative outcome (van 't Klooster et al., 2017a). Meanwhile, van Klink et al. showed that patients with tumor-related epilepsies had favorable outcomes when FR were present within the tumor and peritumoral areas but not outside the resection margin (van Klink et al., 2021). The persistence of FR on post-resection iEcog was associated with poor outcomes.

One fundamental limitation in iEcog analysis is inadequate topographic sampling (e.g., lack of sampling of the bottom of the sulcus cortical dysplasia), resulting in the absence of pre-resection FR (Hussain et al., 2017). Thus, the post-resection FR does not necessarily imply “*de novo*” events; rather, it suggests that the previously undersampled area with FR was exposed following resection (van 't Klooster et al., 2017a). Surgical manipulation does not trigger the emergence of “*de novo*” FR, unlike epileptiform discharges (van Klink et al., 2014). This observation bolsters the case for detecting HFO rather than epileptiform activity and using them for guiding resection.

The multi-center, single-blinded, non-inferiority, randomized controlled trial by Zweiphenning et al. (2022) refuted the previously reported results, showing that the spike-based customized resection outperformed the HFO-based tailored resection in an uncorrected analysis for confounding factors but did not confirm the non-inferiority of HFO. The study findings were inconclusive after accounting for confounding variables. In the subgroup analysis, HFO-based resections were not inferior to spike-based resections in tailored extratemporal lobe cases. The results for the temporal lobe subgroup were inconclusive.

Maccabeo et al. (2022) investigated the role of HFO-based, iEcog-guided resections in patients with neocortical lateral temporal lobe epilepsy, sparing the amygdalo-hippocampal complex. The study did not offer insight into which individuals would benefit from additional HFO-based iEcog-guided amygdalo-hippocampal complex resection. Nonetheless, low post-resection R rates in the mesiotemporal area and older age at epilepsy onset were predictors of a favorable outcome. Patients with poor outcomes had high pre- and post-resection R rates in areas distant from the resection cavity and residual R in the resection cavity. High post-resection R rates in the lateral neocortex were trending toward a favorable outcome. The findings of this study were uninterpretable because of the lack of morphological patterns to distinguish between physiological or pathological R; hence, the authors could not comment on the post-resection R rates in the lateral neocortical areas or foci distant from the resection cavity. They could not confidently assert that these could have been physiological R that emerged after disconnection from the epileptic network. The percentage of HFO removal within the resection cavity was not a relevant predictor of outcome.

Peng et al. (2021) found that HFO resection did not impact outcomes, but their pre-resection within the cortical malformation areas was associated with worse outcomes. Pediatric patients with cortical malformations exhibited a higher pre-resection HFO prevalence in the center of the dysplastic lesion in contrast to tumor cases with peritumoral HFO. One plausible explanation was that the pre-resection FR were indicators of disease severity in patients with focal cortical dysplasia.

The results presented not only highlight the significance of employing more comprehensive and innovative arrays during iEcog to increase the sampling yield and better understand HFO but also analyze the morphological differences between pre- and post-resection R and FR as indicators of the ability of cortical regions to regain functionality (akin to a release phenomenon) following disconnective surgery.

### 5.3 Stimulation induced evoked responses

While most studies have investigated spontaneous HFO, only a handful have examined evoked HFO, triggered by SPES. Single-pulse electric stimulation elicits pathological delayed responses associated with seizure onset zones and physiological early (within 100 ms following stimulation) responses (Mouthaan et al., 2016). Single-pulse electric stimulations induce abrupt synchronization of interconnected neurons, evoking delayed responses that occur later than 100–1,000 ms after stimulation. These responses contain pathological evoked HFO (80–500 Hz) specific to seizure onset zones (Valentín et al., 2002; van 't Klooster et al., 2011; van 't Klooster et al., 2017b; Alarcón et al., 2012).

Mouthaan et al. (2016) analyzed early evoked responses greater than 80 Hz in areas of seizure onset and propagation in 12 patients undergoing iEEG. Early evoked responses were strongly associated with the seizure onset zone as opposed to non-seizure onset zones, separated by several sulci. Stimulation of seizure onset areas evoked early responses in areas involved with seizure propagation. Only one patient had evoked responses in the FR range; all others were in the R range. Contacts with high early evoked response counts in the R band had a high specificity for seizure onset channels. The investigators proposed mapping the epileptic network using both early and delayed responses. This finding contradicted prior knowledge, which suggested that evoked early responses were unrelated to seizure onset zones (Valentín et al., 2002; Valentín et al., 2005a; Valentín et al., 2005b).

van 't Klooster et al. (2017b) reported evoked HFO in 10 patients undergoing iEcog. Two patients without spontaneous FR had evoked FR in the seizure onset zone. Ripples appeared spontaneously in all patients. Ripples were more frequent in functional and electrographically “silent” regions outside the seizure onset zone, irrespective of whether they were evoked or spontaneous, but particularly when evoked. The percentage of resected evoked or spontaneous HFO did not impact the surgical outcome.

Quantitative EEG analysis was employed, utilizing time-frequency information for three frequency ranges (10–80 Hz, 80–250 Hz, and 250–500 Hz) to analyze evoked responses during SPES in 9 patients undergoing iEEG (van 't Klooster et al., 2011). Four patients had a good outcome, while 5 had a poor outcome. Five out of the seven patients with less than 50% of their FR removed had poor outcome, suggesting that time-frequency analysis of the SPES could serve as a new biomarker to identify the epileptogenic cortex. Only two patients, with 94% and 100% removal of the evoked FR area, achieved seizure freedom at 1-year follow-up. Meanwhile, R showed less specificity when identifying seizure onset zones. This suggested that evoked FR were a better marker for the seizure onset zone than R and spikes.

While evidence remains sparse, analyzing evoked HFO is worth exploring. It would provide ancillary data to complement spontaneous HFO and their relationship with the epileptogenic network, seizure onset zones, propagation pathways, and surgical outcomes.

## 6 Evidence from non-invasive monitoring

### 6.1 Scalp EEG

Scalp EEG can detect HFO, particularly in pediatric patients, because of their skull thickness (Zelmann et al., 2014; Shibata and Kobayashi, 2018; Noorlag et al., 2022). Simultaneous scalp and intracranial EEG recordings revealed that HFO detected on the scalp EEG were generated by multiple small cortical sources or varying cortical cluster populations firing HFO asynchronously within a short latency at different foci and generating R on scalp EEG (Zelmann et al., 2014; Kuhnke et al., 2019; Pizzo et al., 2016; Tao et al., 2007; Oishi et al., 2002). Hence, synchronously activated contacts spanning 4–10 cm^2^ would not be necessary to generate focal R on scalp EEG. Furthermore, due to spatial undersampling, iEEG could miss R detected on MEG or high-density (HD) EEG (Zelmann et al., 2014). Nonetheless, scalp EEG may be restricted by myogenic artifact and a low signal-to-noise ratio. The HFO amplitude and rates detected by scalp EEG are lower than those of iEEG. Ripples are detected more frequently than FR on scalp EEG.

Pathological HFO have been identified in premature neonates with seizures and perinatal brain injury given their skull thickness (Noorlag et al., 2021). Nevertheless, the investigators could not demonstrate the predictive value of these HFO in terms of epileptogenesis and outcome.

Klotz et al. (2021) demonstrated that R outperformed spikes and spike R in predicting the development of epilepsy in 56 pediatric patients who experienced their first unprovoked seizures and were followed for 12 months. The R and spike R rates were significantly higher than the spikes in the subgroup of patients who developed epilepsy. Nevertheless, R were the sole event that demonstrated a higher predictive value than the others, making it a more promising potential marker for epileptogenesis on scalp EEG following the first unprovoked seizure in pediatric patients.

Noorlag et al. (2022) conducted a systematic review, revealing that scalp HFO were associated with focal epilepsy, reflected the severity of epilepsy, and were noted in epileptic encephalopathies with impaired cognitive function. Most patients had HFO co-occurring with spikes, and the HFO onset preceded the onset of spikes. A minority displayed HFO overriding spikes. Additionally, spikes exhibited a greater degree of propagation than HFO. The scalp HFO were less sensitive than spikes and gamma frequency activity in localizing the presumed epileptogenic zone. The concordance between the epileptogenic zone and HFO was enhanced through HD-EEG. Poor surgical outcomes were noted in patients with widespread HFO or resection of a smaller percentage of the HFO area.

Tamilia et al. (2020) identified HFO during simultaneous scalp HD-EEG and MEG recording and compared the results to non-simultaneous iEEG findings in pediatric patients undergoing surgical evaluation. There was concordance between R identified on HD-EEG and iEEG. Resection of R overriding spikes, not R without spikes, predicted a good surgical outcome for HD-EEG and MEG. The study confirmed that MEG and HD-EEG could localize with high-precision pathological HFO (R with spikes), whose resection had a higher likelihood of favorable outcomes. Ripples without spikes, unlike R on spikes, were in areas sparing the resection cavity, likely representing physiological events.

The evidence presented above substantiates the utility of scalp and HD-EEG in investigating epileptogenesis and epileptic networks in the pediatric population.

### 6.2 Magnetoencephalography

The utility of MEG in detecting HFO has increased due to its spatial coverage, which is more extensive than that of iEEG sampling (Vasilica et al., 2023; Tamilia et al., 2017). However, the higher rate of artifact and lower signal-to-noise ratio of MEG, compared to iEEG, may limit its ability to explore HFO. Virtual electrodes are better at detecting HFO than physical sensors (Nissen et al., 2016). The number of HFO detected in the center of the seizure onset zone is higher than in the areas farthest from the seizure onset zone when virtual sensors are employed. van Klink et al. adopted virtual electrodes with beamforming techniques in conjunction with HD-EEG and detected more R than the physical sensors, thereby enhancing the localization of the epileptogenic zone (van Klink et al., 2018; van Klink et al., 2019). In contrast to other studies, MEG yielded fewer R than HD-EEG during the simultaneous acquisition of data (Tamilia et al., 2020; Dirodi et al., 2019). Simultaneous scalp EEG and MEG combined with time-frequency maps and isolated islands of R on spectral analysis can exclude artifacts and optimize the yield of recording HFO (Papadelis et al., 2016). When 12 adult and pediatric patients underwent simultaneous SEEG and MEG, the number of HFO detected was not statistically different between the two studies (Vasilica et al., 2023). Nonetheless, MEG showed significantly higher HFO frequencies than SEEG; however, the former displayed poorly shaped HFO compared to SEEG. The concordance of MEG-identified HFO with other invasive and non-invasive modalities has been investigated and correlated with surgical outcomes. Vasillica et al. reported a 58% concordance between SEEG and MEG during simultaneous data acquisition (Vasilica et al., 2023). Seizure freedom was achieved in 85% of patients with concordant MEG and SEEG HFO findings. Okamura et al. demonstrated that 60% of the 15 patients with neocortical lesional epilepsy who underwent resective surgery had concordant equivalent current dipole (ECD) and FR (201–330 Hz) on the gradient magnetic oscillation topography (GMOT) (Okamura et al., 2023). Meanwhile, 87% of the patients had FR around their epileptogenic lesions. The region with the highest power in the FR band on GMOT also displayed a power of >50 Hz on iEEG and iEcog. Four patients with bilaterally scattered ECD were able to achieve lateralization through GMOT analysis in the FR band, implying that these two modalities could be complementary for improved lateralization. The epileptogenic area (determined by iEEG or iEcog) and the area of resection had significantly higher power in the high-frequency band (70–330 Hz) relative to the other areas of the brain.

The spatiotemporal organization of HFO was investigated using MEG. Tamilia et al. examined the spatiotemporal propagation of interictal R in pediatric patients undergoing surgical workup (Tamilia et al., 2021). The investigators identified the R onset zone, defined as the initiating or driving node of the entirety of the R network, and the R zone, encompassing the channels recruited following the onset from a given R onset node. The R onset areas and distribution detected and constructed using virtual MEG sensors were compared to the R onset areas and R zones on the iEEG and HD-EEG. More R propagation was detected on iEEG than on MEG or HD-EEG. Higher amplitude R on IEEG were detected better on MEG and HD-EEG than low amplitude R. The R onset zone was estimated to represent the epileptogenic zone. The resection of the R onset zone resulted in a favorable outcome at the individual level, whereas the resection of the R zone did not impact the outcome. The propagation characteristics of the R zone did not influence the surgical outcome.

Epileptic networks were also analyzed using MEG. Yin et al. demonstrated that patients with insular epilepsy exhibited aberrant effective connectivity in both insular-based networks (involving the insula with seizure onset) and whole-brain connectivity based on interictal HFO with spikes analysis using MEG when compared to health controls (Yin et al., 2020). Aberrant connectivity differed between the left and right insula. Additionally, the authors suggested that HFO-based altered networks could serve as a potential biomarker for investigating epileptic networks in insular epilepsy.

Shi et al. (2023) used MEG to establish that the distribution of R in lateral and medial temporal lobe epilepsies differed significantly from non-temporal lobe epilepsy. The medial temporal lobe had higher R rates than the lateral temporal lobe, followed by the non-temporal lobe. In patients with medial and lateral temporal lobe epilepsies, R were initially observed in the temporal lobe, followed by the parietal and occipital lobes, and finally, in the frontal lobe. Meanwhile, in non-temporal lobe epilepsy, the R clustered in the frontal lobe, followed by the parietal and occipital lobes. Furthermore, the R firing rate varied significantly among these three distinct epilepsy types. Non-temporal epilepsies exhibited significantly higher R channel spatial connectivity than lateral temporal epilepsies, while medial temporal epilepsies had the lowest connectivity. Meanwhile, the medial temporal lobe had a significantly higher clustering coefficient than the lateral temporal lobe, with the non-temporal lobe having the lowest. The highest R ratio was found at the surgical resection site, with a higher degree of centrality. The authors concluded that HFO rates, HFO distribution, spatial connectivity, and clustering coefficient varied substantially among epilepsy types, locations, and pathologies. Moreover, they suggested that R could be pathological biomarkers involved in seizure initiation and propagation based on their distribution pathways.

Magnetoencephalography has also been utilized to study ictal activity and ictal source localization using HFO and correlating them with surgical outcomes. Ramachandrannair et al. studied epileptic spasms in 5 patients, combining MEG, scalp EEG, and iEEG findings acquired non-simultaneously (Ramachandrannair et al., 2008). Although most interictal scalp EEG findings were unilateral, the ictal onset on scalp EEG revealed generalized high-amplitude slow waves superimposed with fast waves, with one patient showing hemispheric electrodectrement. Unilateral interictal MEG spike source (MEGSS) clusters were noted in all patients and overlapped with the ictal onset zone identified on iEEG. Ictal activity on iEEG was typically regional, irrespective of the symmetrical clinical spasms. On iEEG, ictal HFO (150–250 Hz) was regional and brief but sustained and superimposed with slow waves. The clinical onset of spasms was preceded by ictal HFO, with inconsistent spikes, polyspikes, and sharp waves at the HFO onset. The highest HFO occurrence was observed prior to and during spasms. Ictal HFO localized over the Rolandic region in four patients and over the frontal region in one patient. Four patients had additional ictal HFO in contiguous or remote areas. Ictal HFO on iEEG correlated with the MEGSS clusters, with only two patients demonstrating complete overlap. The rest had high concordance rates between MEGSS clusters and ictal HFO on iEEG. Three patients were seizure-free following resection. One patient had >90% and another 50%–75% seizure reduction. The authors suggested that a subset of patients with focal epileptic spasms could be identified and achieve a favorable surgical outcome by employing a similar multimodal approach.

Tenney et al. (2014) observed significant power changes in patients with childhood absence epilepsy at the time of seizure, characterized by 3–4 Hz generalized spike-wave discharges across a range of spectra (1–20 Hz, 20–70 Hz, and 70–150 Hz). High-frequency oscillations were observed at the start, end, or throughout the seizures, as well as inconsistently between patients and within the seizures of the same individual. Gamma frequency band activity was more anteriorly localized in the frontal lobe, lateral prefrontal, and orbitofrontal cortex, whereas the lower frequency bands were observed over the posterior cortex, primarily the parietal lobe and the thalamus. The HFO were restricted to the thalamus and the prefrontal and orbitofrontal cortices. The HFO were less likely to be localized in the parietal, occipital, and temporal regions. Thus, the source localization during absence seizures corroborated the existence of distinct low- and high-frequency bands between the frontal and parietal corticothalamic networks. Whether distinct networks synchronized at the onset of spike-wave discharges remained undetermined.

Given the increasing use of MEG and its optimal spatial coverage, despite its limitations in recording all HFO events, it is essential to integrate this non-invasive modality into the study of HFO in conjunction with iEEG and compare it with areas of ictal onset and propagation, epileptogenic lesions, and surgical outcomes.

### 6.3 Transcranial magnetic stimulation

A few studies have investigated HFO using transcranial magnetic stimulation (TMS). Solomon et al. demonstrated that single-pulse TMS trials at the dorsolateral prefrontal cortex induced theta oscillations in the frontolimbic cortices (precentral, cingulate, and anterolateral frontal) and suppressed higher frequency activity (including at R range) in the frontal (cingulate gyrus), insula, and temporal (superior and middle temporal gyri and hippocampus) areas that were not directly associated with the cortically stimulated sites (Solomon et al., 2024). The investigators concluded that TMS stimulations of specific cortical regions induced changes in neural activity, evoking signals that spread from local to distant brain regions. While the use of TMS remains limited, it would serve as a potential tool in studying connectivity by analyzing high-frequency bands >100 Hz.

## 7 Connectivity analyses using invasive and non-invasive modalities

Earlier, the prevailing perspective was to examine channels with high HFO rates and various brain regions generating HFO at varying frequencies and durations (Jacobs et al., 2012; von Ellenrieder et al., 2017; Gliske et al., 2018; Jacobs et al., 2018a; von Ellenrieder et al., 2016b; Frauscher et al., 2018). Although FR are recognized as biomarkers of epileptogenic tissue, seizure freedom is not always achieved by resecting all the tissues that generate FR (Weiss et al., 2023). Hence, it is crucial to identify other complementary measures to augment the chances of identifying the ictal onset zone and to map the epileptogenic network, the resection of which will lead to a high likelihood of seizure freedom. Amassed evidence has shown that HFO are a component of a local network discharge (Lin et al., 2024). Network-based studies have demonstrated that networks at high-frequency ranges can help comprehend epileptic networks, serving as a reliable tool for assessing seizure onset zones, and epileptogenic zones and predicting surgical outcomes. Epileptic tissue in high-frequency bands (R and FR ranges) can be functionally isolated or disconnected from other brain regions, as revealed by connectivity analyses (Zweiphenning et al., 2016; Zweiphenning et al., 2019; Ibrahim et al., 2012).

Numerous studies have demonstrated that the seizure onset zones were functionally isolated interictally and became more connected during seizure progression (Nissen et al., 2016; Burns et al., 2014). The functional isolation of the channels consisting of gamma-frequency band networks and displaying HFO could be a compensatory mechanism to thwart seizure initiation and halt ictal propagation (van Diessen et al., 2013a; Kramer et al., 2008; Khambhati et al., 2015; Ibrahim et al., 2013). The functional isolation of HFO-generating tissues that included networks with gamma-band frequency, the positive association between networks with FR band eigenvector centrality (EC) and the number of spikes, and the increase in the FR band networks EC in the resected tissue could be a surrogate biomarker for the underlying epileptogenic tissue, generating pathological HFO on a microscale network level that propagated to wider areas during a seizure on a macroscale level (Korzeniewska et al., 2014; Fuertinger et al., 2016). Given the enhanced functional connectivity needed to generate FR, the assumption was that there would be local functional integration of channels covering these areas, at least in the FR band (van Diessen et al., 2013a; Ibrahim et al., 2013). Ictal recordings from iEEG confirmed the increase in synchronization and “hub status” of the epileptogenic tissue (van Mierlo et al., 2013; Varotto et al., 2012; Wilke et al., 2011). Interictally, epileptogenic networks exhibited higher centrality measures in the epileptogenic zone and lower centrality measures in the seizure onset zones (van Diessen et al., 2013a; Varotto et al., 2012; Wilke et al., 2011; van Diessen et al., 2013b; Warren et al., 2010).

Zweiphenning et al. (2016) examined the value of high-frequency network parameters in identifying epileptogenic tissue and guiding resection in patients with temporal lobe epilepsy undergoing iEcog. They investigated areas with HFO and analyzed functional connectivity across all frequency bands during periods of no epileptiform activity. The FR band EC was higher in channels with spikes than without spikes. Gamma-band EC was lower in channels with HFO (R and FR) than in channels without events. The authors displayed functional isolation of networks with gamma-band that showed HFO. Moreover, they demonstrated that focal enhanced connectivity in the channels with FR-band functional networks. This was in accordance with the current observation that FR arose from neuronal clusters that became hyperexcitable due to pathologically reduced interneuron-mediated inhibition and fired synchronously but slightly out of phase. Consequently, resecting areas generating HFO that were functionally isolated in the gamma band would be a possible approach to achieving seizure freedom. The gamma-band functional isolation of that area would reflect the reduced interneuron-mediated inhibition of the HFO-generating tissue.

Samfira et al. (2024) found that connectivity measures were consistent across all channels in the interictal phase, with no hub at any specific frequency, in all pediatric patients with spasms using scalp EEG data. Spatiotemporal dynamic connectivity changes were seen only during clinical spasms. The investigators demonstrated increased connectivity in the FR band (251–400 Hz) at the clinical onset of spasms. During spasms, connectivity in the FR band increased, displaying sustained strengthening in an anteroposterior gradient, maximal in the posterior region (parieto-occipital), reflecting an increase in FR network connectivity following the clinical onset of spasms. The centroparietal area served as the FR network hub based on its maximum closeness centrality. The centroparietal region facilitated information flow within the network, particularly during seizure propagation. The anteroposterior gradients observed in the arousals and awake states of the control group (patients without spasms) were comparable to the posterior > anterior gradient in the closeness centrality during spasms. This finding suggested similarities between the arousal and spasm networks. Additionally, the investigators proposed that the hub channels in the centroparietal region could be surgically targeted to disrupt ictal propagation within the epileptic network based on the connectivity measures.

Ibrahim et al. (2013) investigated the relationship between pathological HFO and the dynamics of network connectivity during seizures. The epileptogenic cortex’s functional connectivity during seizures was frequency-dependent, and the expression of pathological HFO by discrete regions of the epileptic network was associated with specific connectivity patterns. The mean pathological HFO amplitudes of contacts within the seizure onset zone were significantly higher than those outside the seizure onset zone. The seizure onset zone was functionally disconnected at higher frequencies but relatively hyperconnected at lower and slower frequencies. Seizure onset contacts were more deeply embedded in the network (higher clustering coefficient) and more likely to be in network hubs (higher EC) than non-seizure onset contacts at slow frequencies. The seizure onset zone was strongly disconnected from the network at higher frequencies (gamma, R, and FR). At the ictal offset, there was no difference in functional connectivity between the seizure and non-seizure onset areas, implying that loss of disconnection in the fast frequency bands occurred at seizure termination. These findings were concordant with the expanding body of evidence that pathological HFO generation is associated with out-of-phase and aberrant firing of pathological neuronal clusters. This was also congruent with the disconnection of the epileptogenic cortex during ictal onset from surrounding non-epileptogenic tissues to impede ictal propagation (Jiruska et al., 2010; Warren et al., 2010). Hence, the epileptogenic cortex could recruit widespread regions during a seizure via interaction mediated by long-range slow rhythms that are relatively hypersynchronized to the seizure onset zones. Therefore, ictal pathways could be mapped based on the topographic disconnection of the high-frequency activity bands and hyperconnectivity in the delta-theta bands in areas corresponding to brain regions that are identified as ictal onset zones or epileptogenic tissue. Paradoxical hypersynchronization occurred at low frequencies in the seizure onset zone, while concurrent disconnection occurred at higher frequencies in the same zone at seizure onset. It is still uncertain whether HFO are critical components of the epileptic network or byproducts of the epileptic network.

Recent studies have implemented graph-based theoretical analysis to study the spatial geometry of the FR generators in conjunction with defining HFO characteristics to establish the role of HFO hubs and networks in determining the ictal onset zones and predicting surgical outcomes (Weiss et al., 2023). Ibrahim et al. (2013) analyzed ictal connectivity networks at varying frequency bands using graph-based theoretical analysis. At the ictal onset and early propagation, Ibrahim et al. noted a reduction in interregional functional connectivity at frequencies greater than 30 Hz within the epileptogenic tissue and an increased pathological envelope amplitude (i.e., an increase in the tendency toward epileptogenicity). The pathological HFO and the network phase synchrony were interrelated, confirming the frequency-dependent functional connectivity of the epileptogenic tissue and its dynamic changes during ictal onset and propagation.

Stergiadis et al. (2024) utilized graph-based theoretical network measures (betweenness centrality, clustering coefficient, local efficiency, strength, and EC) to investigate the functional connectivity of the iEEG signal in the HFO band to identify biomarkers to map the epileptogenic zone, the resection of which would lead to seizure freedom. Channels with the highest HFO count acted as hubs for the HFO network. The FR-generating tissue displayed significantly high betweenness centrality, EC, and strength, implying the influence of this node in the network and highlighting its hubness. Graph-based results for the FR showed a negative correlation between the local network parameters and the FR rates. The authors posited that the tissue-generating HFO in the FR range was likely extremely hyperexcitable, and as a compensatory mechanism, there was a decrease in specific local metrics to regulate and counteract this aberrant neuronal activity and isolate the FR-generating tissue. They concluded that spatially close nodes are simultaneously connected to each other and to a third node. Accordingly, the FR-generating tissue and seizure onset zone were expected to have a tight interconnection closeness centrality score.

Weiss et al. (2023) used graph-based theoretical measures to demonstrate that the resection ratio of the areas exhibiting FR did not correlate with postoperative seizure freedom. Instead, favorable outcomes were correlated with the FR rate-radius resection difference (RDRRD), a spatial measure of the distance calculated between two contacts occurring at the edge of the resection margin, and the FR mutual information (MI), a temporal measure using the timing of events to measure functional connectivity. These FR network spatiotemporal graph-based theoretical measures improved the accuracy of the predictive value of FR resection.

Lastly, Weiss et al. (2022) demonstrated that non-responders or patients with poor surgical outcomes had decentralized FR networks with widespread, dispersed, highly active, and non-synchronized individual FR-generating nodes. This finding implied that patients would continue to experience seizures even if a portion of the FR network was obliterated or disconnected. A responder or seizure-free patient would likely exhibit very focally connected FR hubs organized in networks within the seizure onset zones, and the resection of these critical and focal HFO hubs would yield seizure freedom. Additionally, including HFO rates within seizure onset zones was insufficient to predict the surgical outcome accurately. Moreover, HFO rates did not differ in determining seizure onset zones between responders and non-responders, except FR > 350 Hz and FR on spikes. In contrast to the low spectral content of FR in the seizure onset zone of the non-responders, the FR rates in the responder group exhibited a higher spectral content in the seizure onset zones. The authors then analyzed RDDRD (which excluded the seizure onset zone) and MI (based on the propagation concept of HFO and long-range synchronization of nodes). These measures distinguished responders from non-responders more accurately when FR > 350 Hz were excluded, as FR > 350 Hz were more often in the seizure onset zone of responders compared to non-seizure onset zones in non-responders. Most non-responders possessed FR networks with a longer characteristic path length, higher nodal strength among non-seizure onset zone nodes relative to seizure onset zone nodes, and lower mean local efficiency in the non-seizure onset zone. This observation corroborated the hypothesis that non-responders had decentralized epileptic networks and asynchronous FR-generating sites that were broadly distributed and consisted of hyperexcitable FR-generating nodes. The FR-generating nodes and seizure onset zones in these non-responders were largely discordant.

The above data corroborate the correlation between the epileptic network, ictal onset areas, and HFO, particularly at FR ranges. The spatiotemporal dynamic nature of HFO is evident in the dynamic changes observed during an ictal event, despite the uncertainty regarding whether they are directly involved in ictal onset, propagation, and termination or are secondary phenomena. The fact that regions with HFO in a specific frequency band are isolated interictally and exhibit increased connectivity at the time of seizure onset merits their investigation as a marker of ictogenesis and the epileptogenic network. In addition, these high-rate HFO-generating areas are isolated from the peripheral tissues as a compensatory mechanism to mitigate their hyperexcitability and aberrant firing. Furthermore, some patients with surgical failure exhibit a decentralized epileptic network with a wide range of FR-generating sites. Therefore, applying connectivity measures is essential in the surgical decision-making process, as they can help clinicians select surgical options, such as resection, thermoablative therapy, or neuromodulation (thalamic *versus* regional).

## 8 High-frequency oscillations and postoperative seizure outcome

The clinical significance of HFO has been assessed through the analysis of postoperative seizure outcomes (Table 1) (Höller et al., 2015; Cho et al., 2014; Akiyama et al., 2011; van 't Klooster et al., 2015; Fujiwara et al., 2012; Cai et al., 2024; van 't Klooster et al., 2017a; Hussain et al., 2017). Several single-center studies have demonstrated concordance of interictal HFO with areas of ictal onset with higher HFO rates in seizure onset areas than non-seizure onset areas (Höller et al., 2015; Hussain et al., 2016; van Klink et al., 2021; Peng et al., 2021; Zweiphenning et al., 2022). Complete removal of the HFO-generating areas resulted in favorable outcomes (Cho et al., 2014; Jacobs et al., 2010; Akiyama et al., 2011; Fujiwara et al., 2012; Wu et al., 2010; van 't Klooster et al., 2017a; Maccabeo et al., 2022; Mouthaan et al., 2016). Haegelen et al. observed that the rates of HFO (R and FR) were higher in areas of seizure onset than in areas outside the seizure onset zone in temporal lobe epilepsy cases but not in extratemporal cases (Haegelen et al., 2013). The resection of these high HFO rate areas in temporal lobe epilepsy cases resulted in favorable surgical outcomes. Results were inconsistent in terms of predictive value of outcome when considering non-rate related HFO characteristics. Grestl et al. demonstrated that HFO alone had better predictive value than spike with overriding HFO and, to a lesser extent, spikes alone (Valentín et al., 2002). Meanwhile Shi et al. showed that ILAE 1 outcome was achieved in patients who had higher mean spike-R rate in the resection cavity than non-removed tissue (van 't Klooster et al., 2011). In neocortical epilepsy, resection of the areas, including FR and R overriding spikes, was associated with favorable outcomes compared to R alone (van 't Klooster et al., 2017b). The results of these single-center studies were refuted in a multi-center study conducted by Jacobs et al. (Sebastian et al., 2023). The authors concluded that HFO were not reliable markers for epileptogenic tissue and possibly were an unreliable predictor of postoperative outcome. However, their analysis had methodological limitations that might have skewed results, including the lack of differentiation between physiologic and pathological HFO.

**TABLE 1:** Summary of studies investigating HFO resection and its correlation with post-operative outcomes.

Study	Sample size	Age (yrs)	Epilepsy etiology	Epilepsy type	EEG MEG	Sampling rate	EEG type	HFO range	Detection	Ictal interictal	Clinical implication	F/u period (months)
Ochi et al. (2007)	9	4–17	NL; heterotopia; CL; FCD	Neocortical	IC	1000 Hz	SD	R	VA	Ictal, pre-ictal	Presence of contacts with high ictal HFO prevalence within the resection area was associated with pos-surgical seizure freedom	11–23
Cho et al. (2014)	15	12–44	NL; cortical dyslamination; FCD; DNET; astrocytoma; HS; gliosis; fibrocalcific neurons	Neocortical	IC	2000 Hz	SD; D	R; FR	AD + post-processing to reject false positives	Interictal	Seizure freedom was significantly associated with resection of areas with high rate HFO.	18–34
Jacobs et al. (2010)	20	21–57	NL; bilateral HS, gliosis; FCD; oligodendroglioma; SWS; neocortical atrophy; TSC; PNH	Mesial temporal; neocortical	IC	2000 Hz	D	R; FR	VA	Interictal	Ratio of removed to non-removed HFO contacts was higher in patients with a good outcome than in those with a poor outcome (significantly higher in R than in FR)	13–37
Akiyama et al. (2011)	28	1–18	NL; gliosis; FCD; MD; TSC; heterotopia; HS; polymicrogyria	Mesial temporal; neocortical	IC	1000 Hz	SD; D	R; FR	AD followed by visual validation	Interictal	Regions exhibiting high R rates were larger than those displaying high FR rates. A more extensive removal of areas with higher FR prevalence was substantially associated with favorable post-operative seizure outcome. A complete resection of areas with high-rate R often improved post-operative outcome	24
van 't Klooster et al. (2015)	54	12–30	HS; tumor with glial component; CM; other	Mesial temporal; neocortical	iEcog	2,048 Hz	SD	R; FR	AD followed by visual validation	Interictal	Residual FR in the post-resection area were associated with seizure recurrence, especially with higher median rate of FR than in patients with good outcome	17–35.8
van Klink et al. (2014)	14	3–37	NL; HS; FCD; TSC; cavernoma; DNET; CM; gangloglioma; astrocytoma; encephalotic cyst	Mesial temporal; neocortical	iEcog	2048 Hz	SD	R; FR	AD followed by visual validation	Interictal	FR, R diminished after resection. Most FR were in resection cavity. Patients with favorable outcome had post-resection R without spikes especially in sensorimotor cortex, implying recovery of physiological function after disconnection from epileptogenic nidus	NA
Fujiwara et al. (2012)	44	9–25	NL; FCD; TSC; gliosis; chronic inflammation	Neocortical	IC	2000 Hz	SD	R; FR	AD	Ictal	Ictal HFO were present in pediatric patients with FCD. Resection of ictal HFO was associated with favorable outcome	NA
Wu et al. (2010)	30	0.67–20.67	NA	Neocortical	iEcog	2000 Hz	SD	FR	VA	Interictal	Complete resection of pre-resection FR was associated with favorable outcome	20–33
González Otárula et al. (2019)	15	16–55	NA	NA	IC	2000 Hz	D	R; FR	Visual marking to direct parameters AD	Interictal	HFO exhibited a network phenomenon feature. FR networks were restricted to seizure onset zones unlike the R networks (source within seizure onset zone). First source channels in network were highest in the HFO rate, hence, resection of source of network did not necessarily determine outcome but rather contact with highest HFO rate	10–77
Thomas et al. (2023)	83	NA	NL; HS; tumor; CL; traumatic brain injuries; FCD	Mesial temporal; neocortical	IC	512 Hz, 1,024 Hz, 2,048 Hz	D	R	AD + VA	Interictal	Seizure freedom was achieved when the ratio of the mean spike-gamma rate in wakefulness was higher in the resected as opposed to the non-resected region. The wakefulness spike-gamma outperformed the R	NA
Tamilia et al. (2018)	20	1.8–17.8	NL; DNET; ganglioglioma; TSC; FCD; encephalomalacia; encephalitis	Neocortical	MEG/HD-EEG; IC	≥600 Hz (IC) 400 Hz (MEG)	HD-EEG; MEG; SD	R	AD	Interictal	Resection of area with ripples-on-spikes predicted good outcome on both HD-EEG and MEG, unlike removal or ripples alone, which had no predictive value	12–48
Cai et al. (2024)	40	14–60	NA	NA	IC	500–2000 Hz	NA	R; FR	AD	Interictal	Favorable surgical outcomes were noted in cases of concordance between HFO-sequences that alighned with the seizure onset zones	>12
van 't Klooster et al. (2017a)	54	15 (M)	HS; glioneural tumors, FCD; TSC, gliosis; cavernoma	Mesial temporal; neocortical	iEcog	2048 Hz	SD	R; FR	AD	Interictal	Persistence of pre-resection FR predicted poor outcome. Pre-resection events without taking any account the post-resection HFO had no impact on outcome	25 (median)
Hussain et al. (2017)	60	2.7–13.4	NA	Neocortical	iEcog	2000 Hz	SD	FR	VA	Interictal	Incomplete resection of FR generating areas was associated with post-operative seizure recurrence or poor outcome	31.2–79.2
Hussain et al. (2016)	30	4.7–13.2	NA	Neocortical	iEcog	2000 Hz	SD	FR	VA	Interictal	Complete FR removal was associated with favorable pos-operative outcome	25.7–79.0
van Klink et al. (2021)	41	0.83–50	Ganglioglioma; DNET; extraventricular neurocytoma; glioma	Mesial temporal; neocortical	iEcog	2048 Hz	SD	R; FR	AD followed by VA	Interictal	FR occurred in higher rates in the tumor and peritumoral tissue. FR outside resection cavity and on post-resection iEcog was associated with poor outcome	29–41
Peng et al. (2021)	34	3–17	FCD; TSC; MD; HS; gliosis; astrocytroma; DNET; ganglioglioma; cavernoma; arachnoid cyst; SWS; benign tumors; pilocytic astrocytoma	Mesial temporal; neocortical	iEcog	800–2048 Hz	SD, D	R	AD	Interictal	R rates were higher in patients with cortical malformation than non-cortical malformation cases. Patients with seizure recurrence had residual pre-resection R in the post-resection cavity	27–80
Zweiphenning et al. (2022)	78	9.2–39	NL; tumor; vascular malformation; HS; FCD; gliosis; TSC	Neocortical	iEcog	2048 Hz	SD	R; FR	VA	Interictal	HFO-guided resection was not non-inferior to spike-guided resection during iEcog In extratemporal cases, HFO showed non-interiority to spikes	12
Maccabeo et al. (2022)	24	3–48	NL; low-grade developmental tumors; cavernoma; encephaloclastic cysts; gliosis; FCD	Lateral temporal neocortical	iEcog	2048 Hz	SD	R; FR	VA	Interictal	Poor outcomes were noted in patients with high rates of pre-resection R and residual R outside the resection margin. Residual FR was also associated with unfavorable outcome	24.3–70.5
van 't Klooster et al. (2017b)	12	8–40	CM; heterotopia; tumor; FCD; HS; TSC; gliosis; encephalomalacia	Neocortical	iEcog	2048 Hz	SD	R; FR	VA	SPES evoked responses-interictal	Evoked HFO, mainly FR, were useful markers for epileptogenic zone and their partial removal could be associated with poor outcome	
Kuhnke et al. (2019)	24	14–63	NL; DNET, cystic lesion; encephalocele; FCD; ganglioglioma; cavernoma, posttraumatic brain injury; gliosis; atrophy; HS	Mesial temporal; neocortical	Scalp; IC	2000 Hz	SD; D	R; FR	VA	Interictal	R and FR were significantly higher in the seizure onset zones than non-seizure onset zones. Rates of HFO were lower in scalp than intracranial EEG. In most patients, scalp HFO occurred in the same area as intracranial HFO. Extent of area on scalp EEG with HFO was broader than in those with poor than good outcome	12
Tamilia et al. (2021)	28	9–16	NL; FCD; HS; tumor; TSC; encephalomalacia; polymicrogyria	Mesial temporal; neocortical	HD-EEG/MEG; IC	MEG (600, 1,000, or 2000 Hz)	HD; SD; D	R	AD	Interictal	Virtual R onset zone was closer to resection in patients with good outcome than poor outcome. Percentage of R onset zone/R zone resection was higher in good than poor outcome patients Resection of virtual R onset zone and not the virtual R zone predicted good outcome	13–36.5
Jacobs et al. (2018a)	52	0.5–62	NL; Encephalocele, FCD, HS, DNET, Gliosis, TSC; atrophy; Rasmussen’s encephalitis; cyst; polymicrogyria	Mesial temporal; neocortical	IC	2000 Hz	SD; D	R; FR	Initial brief visual validation of AD then remaining all AD	Interictal	Resection of HFO generating tissue did not reliably predict post-surgical outcome at an individual level. At a group level, removal of HFO generating area was correlated with seizure free outcome	12
Weiss et al. (2023)	23	18–70	NL; encephalomalacia; HS; PNH; prior ATL; prior resection; arachnoid cyst; temporal FLAIR hyperintense signal and mild enhancement; extra-temporal T2 FLAIR hyperintense signal	Mesial temporal; neocortical	IC	2000 Hz	D	FR	AD; graph-based theoratical models	Interictal	FR resection ratio did not correlate with postoperative outcome; the FR (especially >350 Hz) rate- distance difference metric was significantly correlated with post-operative outcome	0.2–60
Lin et al. (2024)	28	5–59	FCD; gliosis; HS; glioma; DNET; tumor-NFI	Mesial temporal; neocortical	IC	4,096 Hz	SD; D	R; FR	AD	Interictal	HFO were demonstrated to be as part of a network event and not solitary events. Incorporating network features, such as centrality index and spatio-temporal pattern of HFO, was more predictive of seizure outcome than HFO rate alone	NA
Haegelen et al. (2013)	30	16–58	HS; polymicrogyria; hippocampal atrophy Temporal cyst; post-resection cavities; cortical tubers; arachnoid cyst; porencephalic cyst; PNH; meningioma; FCD	Neocortical	IC	2000 Hz	SD; D	R; FR	VA	Interictal	Surgical outcome was better in the whole group and temporal lobe epilepsy patients but not the extratemporal lobe epilepsy cases where high rates of HFO were included in resection cavity	9–72
Kerber et al. (2013)	22	8–57	FCD	Neocortical	IC	1024 Hz	SD; D	R; FR	VA + AD	Interictal	R and FR were significantly higher in areas of seizure onset. In seizure-free patients, the resected areas had significantly higher rates of FR compared to other brain areas	>12 months
Okanishi et al. (2014)	10	2.7–18.4	TSC	Neocortical	IC	2000 Hz	SD; D	R; FR	AD + VA	Interictal	Patients with multiple tubers and seizure onset zones had also multiple foci of high-rate R and FR. Resection of areas with high-occurrence rates of R and FR was associated with good outcome	19–76
Weiss et al. (2015)	46	9–54	NL; HS; cavernoma; gliosis; pilocytic astrocytoma; DNET; MD; FCD; chronic vascular changes; hyaline astrocytopathy	Mesial temporal; neocortical	IC	500–1000 Hz	SD; D	High gamma (80–150 Hz)	VA + AD	Ictal	Extent of resection of seizure onset zone and the early sites that display phase-locked high gamma activity is associated with post-operative outcome	0.75–6.5
Li et al. (2021c)	16	3–54	Gliosis; HS; FCD	Mesial temporal; neocortical	IC	512–1024 Hz	D	30–150 Hz	Graph measures	Ictal	Resection of critical hubs (high frequency) was associated with favorable outcomes. High frequency hubs were significantly higher in resected areas and were noted in the early/middle part of the ictal activity. Low frequency hubs emerged in the late part of the ictal activity	NA
Fedele et al. (2017)	20	17–52	FCD; Gliosis; HS; glioma; ganglioglioma; cavernoma	Mesial temporal; neocortical	IC	4000 Hz	SD; D	R; FR	AD + VA	Interictal	Resection of the HFO area (high-rate R co-occurring with FR) was associated with post-operative favorable outcome	10–46
Fedele et al. (2016)	14	NA	NA	NA	iEcog	2048 Hz	SD	R; FR	AD + VA	Interictal	FR identified by AD post-resection retrospectively were able to correlate with poor surgical outcome	>12

AD, automated detector; ATL, anterior temporal lobectomy; CL, cystic lesion; CM, cortical malformation; D, depth; DNET, dysembryoplastic neuroepithelial tumor; FCD, focal cortical dysplasia; FR, fast ripple; F/u, Follow-up; HD, High-density; HS, hippocampal sclerosis; IC, intracranial recording; iEcog, Intraoperative electrocorticography; M, median; MD, microdysgenesis; NA, not available; NL, Non-lesional; PNH, periventricular nodular heterotopia; R, ripple; SD, subdural; SEEG, stereoelectroencephalography; SPES, single pulse electrical stimulation; SWS: sturge weber syndrome; TSC, tubers due to tuberous sclerosis complex; VA, visual annotation; YRS, years.

*Neocortical, Extra-temporal and temporal.

The results of numerous systematic reviews and meta-analyses have been inconsistent. Wang et al. conducted a systematic review and meta-analysis derived from 47 studies including 1,026 patients with R and FR (Wang et al., 2024). Patients who underwent complete resection of the HFO regions (both R and FR) exhibited a greater likelihood of achieving seizure freedom compared to those who did not have complete removal of HFO. In patients undergoing iEcog-guided tailored resection, complete FR removal demonstrated greater efficacy in achieving seizure freedom in cases of extra-temporal lobe epilepsy compared to those with temporal lobe epilepsy. Additionally, patients with lesional MRI findings and complete resection of focal regions exhibited a higher likelihood of achieving seizure freedom compared to those with non-lesional cases. Qu et al. (2024) demonstrated that the complete removal of R and FR-generating tissues was significantly better than incomplete removal. This was also the case for partial removal, which outperformed no removal of HFO areas. However, the authors did not factor in the spatiotemporal extent of the resected HFO (their location outside the area of ictal onset or the association with an MRI lesion), which is essential to study HFO as an electrophysiological biomarker that can map the boundaries of the epileptic network rather than merely identifying the “sampled” ictal onset area. The findings of Qu et al.’s meta-analysis were contrary to Gloss et al.’s Cochrane review, which found that HFO resection did not impact surgical outcomes (Gloss et al., 2017). Wang et al. (2024) displayed that favorable surgical outcomes were attained more often with complete resection (at least ≥80%) of areas generating HFO than with incomplete resection of HFO areas, similar to Qu et al. (2024) findings. Furthermore, the complete removal of FR areas was better at achieving favorable outcomes in patients with extra-temporal lobe epilepsy than in those with temporal lobe epilepsy. Nonetheless, this analysis had several limitations, including the absence of a consistent definition of “resection area,” the ambiguity regarding the location of the resected HFO area or contacts (e.g., contiguous *versus* non-contiguous contacts) concerning the area of ictal onset, the extent of the resection area and its congruence with the area of ictal onset and early propagation, and the topographic distribution of HFO and their organization into a consistent hierarchical distribution. Höller et al. (2015) meta-analysis showed that the frequency of the HFO rather than the number of channels displaying HFO were related to surgical outcome.

It is conceivable that HFO “hubs” exist and maintain synaptic and direct connections to remote HFO sites (van Diessen et al., 2013b). Through low-frequency EEG rhythms (e.g., delta or theta waveforms), these interconnected sites can coordinate activity with HFO hubs. Consequently, severe epilepsy forms may have multiple HFO hubs implying strong modulatory effect on remote area. This implies that resection of all the HFO hubs or their neuromodulation will be associated with high likelihood of seizure freedom (Zweiphenning et al., 2019).

van 't Klooster et al. (2015) found that patients with focal refractory epilepsy who underwent resection had recurrent postoperative seizures when residual FR were noted on iEcog. The authors did not mention the association of the residual FR with the resected HFO. Had residual FR in the resection margins been synchronous with the resected FR, this would have substantiated the hypothesis that HFO with spatiotemporal organization, otherwise described as “propagation” or “network,” mirrored the epileptic network. It would also have questioned whether extending resection to include residual FR in the resection margin would have improved the outcome. The absence of residual post-resection FR in patients with recurrent seizures was another unexplained finding. The authors observed Engel 3 and 4 outcomes in patients with a high number and broad area of FR, substantiating question of the extent of FR resection areas (Cho et al., 2014; van 't Klooster et al., 2015; Kerber et al., 2013; Okanishi et al., 2014; Bragin et al., 2003; Zijlmans et al., 2009).

Okanishi et al. (2014) demonstrated that patients with tuberous sclerosis complex and multiple tubers had multiple segments (referring to contiguous channels) of seizure onset contacts, as well as high rates of interictal R and FR. These segments showed a wide distribution of HFO across multiple lobes (e.g., temporo-parieto-occipital) and epileptic networks. Resection of areas with high R and FR occurrence rates predicted outcomes more accurately (statistically significant) than the resection ratio of contacts involved in seizure onset. Only 3 out of the 10 patients attained Engel I outcomes, and the remaining attained Engel II-IV outcomes. The study did not elaborate on the characteristics of the “HFO segments,” their spatiotemporal extent, or their correlation with the entirety of the epileptic network (onset and propagation). It also did not explicitly define the concordance between high HFO occurrence and the seizure onset zone. The authors suggested that, in this context, high HFO occurrence would be a better surrogate marker of the epileptic network than the seizure onset zone.

Recent studies looked at combined HFO rates and HFO network measures to predict surgical outcomes. Lin et al. (2024) demonstrated that HFO are not isolated events but components of a local epileptiform network. Including the HFO rate-based seizure onset predictive measures led to a trend toward a better outcome but did not achieve statistical significance. To enhance the likelihood of accurately predicting a favorable surgical outcome, the authors combined HFO network properties with HFO rate analysis and the seizure onset zone. Consequently, the probability of a positive outcome was increased for patients who underwent palliative surgery (resection of less than 80% of the seizure onset zone) when the HFO network analysis was incorporated into the HFO rate. Furthermore, the authors acknowledged that network analysis of HFO can be challenging due to the variability of HFO detections. If an HFO is detected on one channel, there might be no detections on the immediate neighboring channels.

Tamilia et al. (2018) demonstrated the spatiotemporal propagation and hierarchical organization of R into onset and propagation zones (the appearance of R on subsequent contact with a lag) in pediatric patients. The mean rate of R onset contacts within the resection cavity and their removal was associated with a favorable outcome. The postoperative outcome was not affected by the R spread zone, isolated R contacts (lacking propagation), or any spike zone (spike source contact and its propagation contacts). The authors proposed that the R onset nodes could serve as a potential biomarker for epileptogenic tissue and a predictor of surgical outcomes in pediatric patients. Their findings confirmed that the areas of R onset were more epileptogenic than the R spread zone.

Some explored coupling of HFO with lower-frequency oscillations and its impact on surgical outcome. The resection of areas revealing coupling of HFO and low-frequency oscillations during seizures or sleep could yield favorable outcomes (von Ellenrieder et al., 2016b; Iimura et al., 2018; Nonoda et al., 2016; Motoi et al., 2018; Amiri et al., 2019; Weiss et al., 2015). Cross-frequency coupling between pathological HFO and theta-alpha rhythms was significantly elevated in the seizure onset zone than in non-epileptic regions (Ibrahim et al., 2014). Early phase-locked high-frequency gamma activity during the ictal phase could be associated with a favorable postoperative outcome (Weiss et al., 2015). In the meantime, delta-modulated coupling to HFO was essential in identifying the epileptogenic zone in neocortical extratemporal lobe epilepsy (Guirgis et al., 2015). This analysis was also applied to scalp EEG, where cross-frequency coupling predicted seizure onset zones and localized the epileptogenic source (Li et al., 2016; Jacobs D. et al., 2018). As a result, determining HFO activity in conjunction with low-frequency activity was a more effective way to investigate ictal onset areas. However, the high-frequency hubs were involved in initiating seizures, not the low-frequency hubs, and predicted surgical outcomes (Li C. et al., 2021). The high-frequency hubs localized to the resected channels and emerged several seconds after seizure onset. The outcome would be poor if the high-frequency hubs were widely distributed and outside the resection at an early age. During the early and middle stages of the seizure, the high-frequency hub had significantly higher strength in the resected regions than in the non-resected areas. As for the low-frequency hub, increased strength inside the resected region at a later seizure stage was associated with Engel I. The early transition from pathological high-frequency hubs to the later emergence of low-frequency hubs was displayed in cross-frequency coupled networks. Stronger low-frequency and high-frequency hubs within the resection region had been associated with favorable outcomes.

The previous results suggest a correlation between HFO and surgical outcomes; however, the methodological variation in these events and their acquisition modality (e.g., invasive *versus* non-invasive or subdural *versus* depth contacts) renders a comparative analysis challenging. Additionally, the evidence favors the investigation of HFO as a network phenomenon rather than isolated events, integrating the analysis of cross-coupling of frequencies that are lower in range than the HFO range as well as connectivity measures as described in the prior section.

## 9 Challenges and future perspectives

**FIGURE 1:**
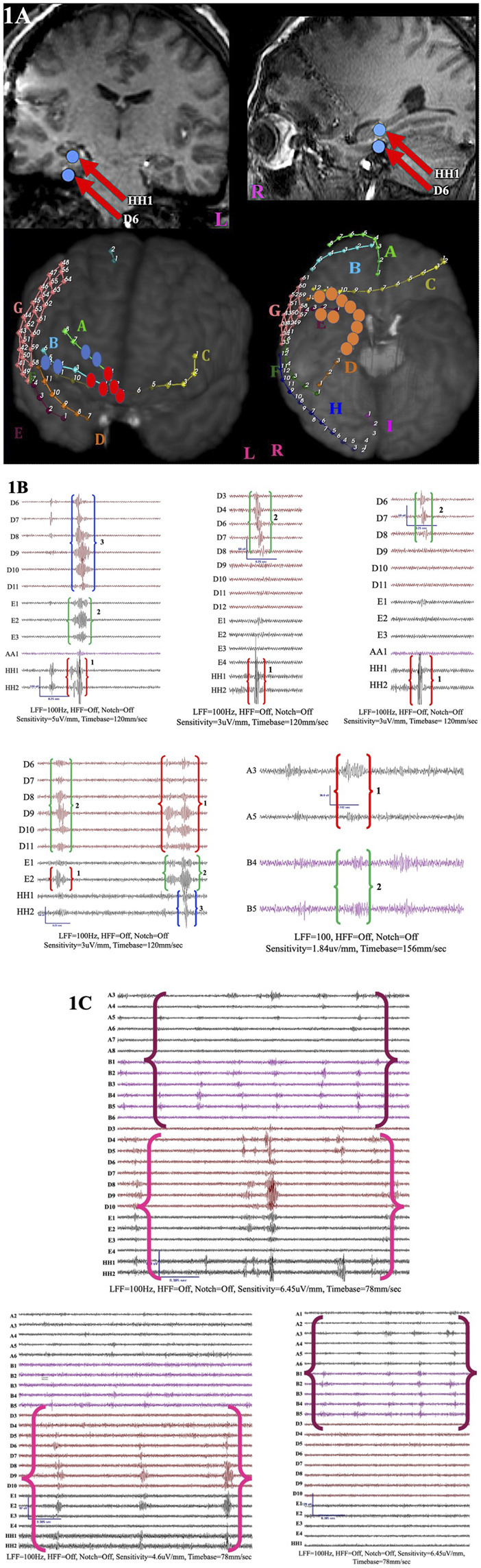
An illustration of the spatiotemporal organization of the HFO network. **(A)** Demonstration of intracranial macroelectrode contacts as part of the surgical workup in a patient with non-lesional pharmacoresistant epilepsy of unknown etiology and clinical semiology lateralizing and localizing to the right frontal and temporal areas. First row: MRI brain showing the implanted intracranial contacts. The red arrows indicate foci consistently involved in the HFO (R range) network, predominantly at the onset of the HFO network sequence. Throughout the recording, the HH and D contacts were predominantly synchronous. Second row: MRI reconstructions show R-range HFO networks primarily found in the frontal lobe (blue and red) and the base of the temporal lobe (orange). The blue contacts represent the most common contacts within the HFO network, which occur concurrently with the basal temporal areas. The red dots indicate a second network population in the anterior frontal lobe **(B)**. **(B)** Five illustrative images depict the two HFO network sequences consistently present throughout the recording period. In network one, the contacts were HH1-2, D3-11, and EE1-3. The precedence of one *versus* the other contacts was variable, but the contact that emerged first in the sequence was consistently HH1/HH2. The contacts in the second network were A3/A5 and B4/B5. The red brackets denote the first channels in the sequence (numbered 1); the green brackets represent the second channels in the sequence (numbered 2); and the blue brackets represent the last channels in the sequence (numbered 3). **(C)** Both networks (one defined by magenta brackets and two by purple brackets) occurred simultaneously and independently. It is unclear if the concurrent occurrence was coincidental or if a single mechanism triggered the two networks simultaneously.

Identifying a reliable surrogate biomarker for epileptogenic networks is crucial, notably since the incidence of surgical failures has not diminished over time. High-frequency oscillations are a potential biomarker for epileptogenic networks, and additional rigorous investigation is warranted. It is evident that HFO are not isolated occurrences; they have hierarchical organization and exhibit spatiotemporal variations (Figure 1). Hence, it is worthwhile investigating HFO on a network scale to tackle surgical failures, particularly in challenging scenarios such as non-lesional and poorly localized epilepsies.

The latest innovative tools, such as optogenetic and chemogenic techniques, can be used to explore the spatiotemporal dynamics of dysfunctional neuronal activity and PIN involved in generating HFO. Chvojka et al. (2024) utilized optogenetics to study HFO in a murine model of neocortical epilepsy with focal cortical dysplasia (FCD) type II. Pathological HFO (up to 800 Hz) were generated by the FCD and propagated outside the lesion. Foci in the contralateral hemisphere also generated HFO independent of the FCD lesion, implying widespread network dysfunction in patients with FCD. The neurons carrying the mTOR mutation and expressing channelrhodopsin-2 represented functionally interconnected and active FCD network components (Jiruska et al., 2017; Chvojka et al., 2024; Ibarz et al., 2010). This novel approach was intriguing because it localized the epileptogenic or seizure-generating tissue and mapped the HFO networks, which were spatially not limited to the seizure onset zone and the FCD lesion but extended to include distant non-lesional regions in the ipsilateral and contralateral hemispheres.

However, there are still significant obstacles to studying HFO. These factors include the absence of consensus in definitions (such as morphology, amplitude, duration, frequency, interevent intervals), terminologies (such as cluster, propagation, sequence, zone), modalities used (MEG, iEcog, scalp EEG, SEEG *versus* subdural contacts), analysis methodologies (rates, graph based theoretical analysis), sampling rates, features that differentiate between physiologic and pathological R and FR to avoid any bias in analysis, variability in the automated detectors and their set threshold for detections that increase rates of false positive detections, undersampling in select study types, and the need for further improvement of other non-invasive modalities, as well as the time-consuming nature of visual analysis.
